# Designing of Highly Effective Complementary and Mismatch siRNAs for Silencing a Gene

**DOI:** 10.1371/journal.pone.0023443

**Published:** 2011-08-10

**Authors:** Firoz Ahmed, Gajendra P. S. Raghava

**Affiliations:** Bioinformatics Centre, Institute of Microbial Technology, Chandigarh, India; Victor Chang Cardiac Research Institute (VCCRI), Australia

## Abstract

In past, numerous methods have been developed for predicting efficacy of short interfering RNA (siRNA). However these methods have been developed for predicting efficacy of fully complementary siRNA against a gene. Best of author's knowledge no method has been developed for predicting efficacy of mismatch siRNA against a gene. In this study, a systematic attempt has been made to identify highly effective complementary as well as mismatch siRNAs for silencing a gene.

Support vector machine (SVM) based models have been developed for predicting efficacy of siRNAs using composition, binary and hybrid pattern siRNAs. We achieved maximum correlation 0.67 between predicted and actual efficacy of siRNAs using hybrid model. All models were trained and tested on a dataset of 2182 siRNAs and performance was evaluated using five-fold cross validation techniques. The performance of our method desiRm is comparable to other well-known methods. In this study, first time attempt has been made to design mutant siRNAs (mismatch siRNAs). In this approach we mutated a given siRNA on all possible sites/positions with all possible nucleotides. Efficacy of each mutated siRNA is predicted using our method desiRm. It is well known from literature that mismatches between siRNA and target affects the silencing efficacy. Thus we have incorporated the rules derived from base mismatches experimental data to find out over all efficacy of mutated or mismatch siRNAs. Finally we developed a webserver, desiRm (http://www.imtech.res.in/raghava/desirm/) for designing highly effective siRNA for silencing a gene. This tool will be helpful to design siRNA to degrade disease isoform of heterozygous single nucleotide polymorphism gene without depleting the wild type protein.

## Introduction

RNA interference (RNAi) is a natural mechanism evolved in complex organisms to regulate the gene expression. This mechanism also provide defense against viruses and transposable material to maintain the genome integrity [1]. There has been increasing interest to harness this mechanism to silence a specific mRNA. RNAi is triggered whenever a cell encounter long dsRNA molecules and subsequently cleave them into small interfering RNAs (siRNAs) using Dicer enzyme. siRNA is ∼21 nucleotide (nt) long dsRNA having 2 nt overhang on 3′-end. Afterward, siRNA unwound and one strand associated with nuclease-containing protein complex (RISC). Subsequently RISC containing siRNA bind to the complementary mRNA and promotes cleavage/degradation of mRNA [2].

siRNAs have become an important tool for silencing gene of interest and have emerging as potential therapeutics. The beauty of the system that makes it a powerful tool lies in sequence specificity towards particular gene, its quick effect, and cost effectiveness. Importantly, it makes feasible for large-scale functional genomics studies. It has been shown that knockdown effect (efficacy) of siRNA is varying according to target site on mRNA and hence, very limited set of siRNAs show high efficacy [3]. Huesken *et al.* analyzed experimental data to understand relationship between the siRNA sequence and its silencing effect on 34 mRNA species [4]. They also developed an Artificial Neural Network (ANN) based method BIOPREDsi and achieved maximum correlation 0.66 between actual and predicted efficacy [4]. In past, number of methods have been developed for predicting efficacy of siRNA [5], [6], [7], [8], [9]. In a recent study, performance of various methods have been evaluated which showed BIOPREDsi, ThermoComposition21 and DSIR are highly accurate and reliable methods [8], [10].

Initially it was believed that full complementary siRNA is needed to silence a target gene. However, studies have shown that siRNA behaves like miRNA and suppress protein synthesis when it is not fully complementary to the target, indicating mismatches are allowed during target selection by siRNA [2], [11]. This phenomenon also raised very important problem about off-target effect where unintended target genes suppressed by siRNA [12], [13], [14]. A study indicates that seed region of siRNA, 2-8 nt from 5′-end, is important for target finding and single mismatch within seed region can change the off-target transcripts without effecting silencing efficiency of original target transcript [14]. Initially off-target sequences were searched using similarity based methods against mRNA sequence database but the strategy was not successful due to lack of knowledge about level of sequence similarity required for off-target effect. To understand the silencing effect of mismatch between siRNA and target, several studies were conducted [15], [16], [17], [18], [19], [20], [21]. The study by Du *et al.* reveals position of the mismatch generated in the target influence silencing and categorized them as; (a) High tolerance: mismatch at position 1, 2, 18, or 19, which does not affect the efficacy. (b) Low tolerance: mismatch at position 5-11 which results into abolishing the RNAi activity and remain position is (c) of moderate tolerance [15]. It also showed the impact of mismatched nucleotide and found A:C and G:U are well tolerated mismatch. Furthermore the silencing effect of double-nucleotide mismatches were also studied [17]. Recently, a very systematic study was conducted by using 20 siRNAs against 400 various mismatched targets to generate a model for single nucleotide-mismatch [21]. This study analyzed all combinations of mismatched siRNA:target and demonstrated that efficacy can be influenced by position and type of nucleotide mismatched. The work also demonstrated that most tolerant mismatch was A:C while least one was A:G in term of siRNA:target. It was observed that swapping of mismatched nucleotides at some position dramatically changed the efficacy *e.g.* at position 17 of siRNA both A:C and C:A mismatched are well tolerated while at position 12 only A:C mismatch is tolerated not C:A. However, study also demonstrated the importance of creating mismatch between sense and antisense strand of siRNA in order to make more asymmetric siRNA which leads to improve silencing efficacy [20]. In order to find off target sequence, methods has been developed which incorporate features like seed complementary region and nucleotide mismatch to predict potential off-targets [22]. To the best of author's knowledge, lack of specificity of siRNA is considered as major drawback in designing any siRNA based therapy.

Investigation indicates that a large portion in mRNA could not be targeted for siRNA because of having low efficacy [3]. Thus, it makes limited choice for selecting target site. Furthermore, the requirement to enhance efficacy of a siRNA against particular target site is not fulfilled by available methods. In this study, we have examined whether weakness of siRNA (poor specificity) can be exploited to design mutant siRNA of desired efficacy. It is well known that all siRNAs is not equally effective even if they are fully complementary to mRNA. On the other side, we also know from experimental studies that few mismatches at specific position can be tolerated. Based on this hypothesis a prediction method has been developed for designing effective mismatch siRNA against mRNA.

This study having two sections: (1) The development of a model for predicting siRNA efficacy, and (2) The creation of mutation in the siRNA sequence to enhance its efficacy. This facility is accessible to scientific community through web based portal at http://www.imtech.res.in/raghava/desirm/.

## Methods

### Datasets

The main dataset used in this study contains 2182 siRNAs. All models trained, tested and evaluated using five-fold cross-validation techniques on main dataset. This dataset was obtained from Huesken *et al.* [4] and have been used for developing number of existing methods. In order to compare performance of our method with existing methods, we obtained benchmarking data from Ichihara *et al.* [8]. This benchmarking data contains two datasets; I) training dataset having 2431 siRNAs [consist of 2182 (main dataset) + 249 (testing dataset)] taken from [4] and ii) testing dataset consists of 419 siRNAs [23], [24], [25], [26], [27].

### Features used for models development

#### Composition based features

Nucleotide composition: The nucleotide composition determines the occurrences of different types of nucleotides, dinucleotides, trinucleotide etc. We compute mono-, di,- tri-, and tetra-nucleotide composition of siRNAs that generate vector of 4 (A, C, G, and U), 16 (AA, AC, AG, CG, AU,…, UU), 64 (AAA, AAC, AAG,…, UUU), and 256 (AAAA, AAAC, AAAG,…,UUUU) respectively.

Split nucleotide composition: In this case whole sequence was divided into two equal parts and nucleotide composition of each part is calculated separately. Composition of both part is used to develop our models, in this case dimension of input vector was doubled [28]. For instance 21 nt sequence was divided into nearly half 11 nt and 11 nt, mononucleotide composition was calculated for each part and combine to form vector dimensions of 8.

Higher order nucleotide composition: In simple dinucleotide composition we considered local order (1^st^ order) where interaction between i^th^ and (i + 1)^th^ nucleotide is taken into account. In case of second order dinucleotide composition, interaction of 1^st^ with 3^rd^ nucleotide is considered *i.e.* i^th^ and (i + 2)^th^. Similarly in case of third order dinucleotide composition interaction of 1^st^ with 4^th^ nucleotide is considered.

#### Position specific features

Binary pattern of nucleotides: This gives information about occurrences of position specific nucleotide in siRNA sequence. In this case each nucleotide was represented by binary pattern of dimensions four (A by [1,0,0,0], C by [0,1,0,0], G by [0,0,1,0] and U by [0,0,0,1]). Thus, a sequence of 21 nucleotides of miRNA was represented by a vector of dimensions 84 (4×21).

Binary pattern of dinucleotides: Instead of considering one nucleotide as in binary pattern, occurrence of two consecutive nucleotides at particular position was considered.

Binary of condense: Sequence was divided into two equal parts and binary pattern of both part were calculated and merged into each other (like hairpin structure) so that 5′-end and 3′-end of a sequence are at same position.

Hydrogen bond: The hydrogen bonding properties were depicted as “3” for G and C while “2” was assigned in case of A and U.

Thermodynamic: The value of thermodynamic propertied at each position were taken from [10].

Target site accessibility: Target site accessibility in terms of probability of being unpaired is calculated using RNAplfold [29]. We used parameter (W = 80, L = 40, u = 16) for calculating target site accessibility which was considered as the best parameters for differentiating between functional and non-functional siRNA [6].

Scaling of feature: During hybrid approach various different features were considered at a time creating a large range of feature values that resulted into the poor performance of models [30]. Hence we normalized the values in the range of 1–10 using scaling feature of libSVM software (http://www.csie.ntu.edu.tw/~cjlin/papers/guide/guide.pdf).

#### Prediction approaches

In order to develop models for siRNA efficacy prediction, various features of siRNAs were used. SVM^light^ [31], was implemented for models development.

#### Performance measures

In order to evaluate performance of our models, we used following standard parameters; 1) correlation coefficient (R), II) coefficient of determination (R^2^), III) mean absolute error (MAE), root mean squared error (RMSE). All models were evaluated using five-fold cross validation technique.
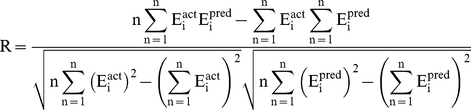


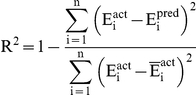


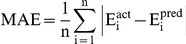


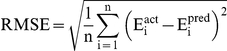



Where n is the size of test set, E_i_^pred^, and E_i_^act^ is the predicted and actual efficacy respectively. 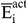 is the average of actual efficacy in test set.

## Results

All models were trained and tested on main datasets consist of 2182 siRNAs where each siRNA is 21 nucleotides long. In this strategy dataset was randomly divided into five sets, four sets were used for training and remaining set for testing. This process is repeated five times so that each set is used once for testing.

### Composition based models

First, SVM based models have been developed using different types of nucleotide compositions and obtained maximum correlation of 0.574 between predicted and actual efficacy using tetranucleotide composition. The performance of all composition-based models has been shown in Table 1. Similarly, SVM based models have been developed using split nucleotide composition and achieve maximum correlation of 0.508 using trinucleotide composition. Finally models were developed using higher order nucleotide composition and achieved best correlation 0.579 between predicted and actual efficacy. In comparison to simple trinucleotide composition (R = 0.574), substantial increase in efficacy was observed using 2^nd^ order trinucleotide composition reveals the importance of pattern of nucleotides and influence of single gap on efficacy (Table 1).

**Table 1:** Performance of SVM-based models for siRNA efficacy prediction developed using composition based features.

Composition	Features	Vector	R	R2	MAE	RMSE	g	c	j
**Nucleotide Composition**	Mono	4	0.316	0.095	0.152	0.190	0.001	1	1
	Di	16	0.450	0.145	0.145	0.185	0.001	3	2
	Tri	64	0.515	0.248	0.138	0.173	0.001	1	2
	Tetra	256	0.574	0.312	0.131	0.166	0.0001	10	2
**Split nucleotide composition**	Mono	8	0.355	-0.03	0.161	0.203	0.001	1	3
	Di	32	0.453	0.203	0.143	0.178	0.001	1	3
	Tri	128	0.508	0.243	0.137	0.174	0.0001	2	2
**Higher order composition**	2^nd^ order Di	16	0.420	0.115	0.149	0.188	0.001	1	2
	3^rd^ order Di	16	0.467	0.207	0.143	0.178	0.001	1	1
	4^th^ order Di	16	0.461	0.150	0.146	0.184	0.001	1	2
	**2^nd^ order Tri**	**64**	**0.579**	**0.332**	**0.128**	**0.163**	**0.001**	**1**	**1**
	3^rd^ order Tri	64	0.483	0.218	0.141	0.177	0.001	1	1
	2^nd^ order Tetra	256	0.502	0.222	0.139	0.176	0.0001	10	2

Mono: mononucleotide; di: dinucleotide; tri: trinucleotide; tetra: tetranucleotide; R: correlation coefficiet; R2: Coefficient of determination; MAE: Mean absolute error; RMSE: Root mean square of error; g, c, and j are SVM parameters.

### Models based on position specific features

One of major disadvantage of above composition based models is that they used only frequency of different types of nucleotides and hence do not consider the information about position of nucleotides in siRNA. In order to overcome this problem we created binary patterns for siRNA, which provide complete information (position and type of nucleotide). First SVM based model was developed using binary pattern of nucleotide composition and achieve correlation coefficient of 0.637 (Table 2). This model outperforms all the models based on composition, which indicate importance of position of nucleotides in siRNA. As shown in Table 2, SVM models were developed using various types of binary patterns like dinucleotide, hydrogen bond. However, we got maximum performance using binary pattern of nucleotides.

**Table 2:** Performance of SVM-based model for siRNA efficacy prediction developed using position specific feature and our method desiRm.

Features	Vector	R	R2	MAE	RMSE	g	c	j
Binary pattern	84	0.637	0.406	0.122	0.154	0.01	1	1
Binary of di	320	0.563	0.272	0.135	0.170	0.001	6	2
Binary of Condense	40	0.449	0.200	0.142	0.179	0.001	10	1
AU, GC	42	0.362	0.130	0.149	0.186	0.001	1	1
Hydrogen bond	21	0.579	0.335	0.130	0.163	0.01	2	1
Thermodynamics	19	0.577	0.332	0.129	0.163	0.001	10	1
**desiRm^21^**	**168**	**0.670**	**0.448**	**0.118**	**0.148**	**0.001**	**2**	**1**

### Hybrid models

In this study we developed models using two or more than two types of features and called Hybrid models. First hybrid models were developed using composition based features where two or more than two types of compositions were used for developing models (Table S1). Similarly, we developed hybrid models using position specific features; we found binary pattern and thermodynamics achieved better performance (Table S1). We also developed hybrid models using percent nucleotide composition, nucleotide frequency and binary pattern as input feature. Finally, we achieved highest correlation coefficient of 0.670 by using our hybrid model, which uses nucleotide frequency and position specific based features (Mono+Di+Tri+Binary pattern). We called this model desiRm^21^ in this study (Table 2).

### Comparison with existing methods

It is important to compare performance of newly developed method with existing methods. In order to compare any two methods, one should use same dataset for training and testing. Recently, Ichihara *et al.* [8] compare performance of major existing methods. In this study we used same data for evaluating performance of our newly developed method desiRm^21^. We trained our model on 2431 siRNAs and tested on 419 siRNAs. As shown in Table 3, performance of desiRm^21^ is comparable to previously developed methods.

**Table 3:** Performance of desiRm^21^ and other four algorithms on test dataset containing 419 siRNA.

Methods	R	R2	MAE	RMSE
i-Score	0.557	0.217	0.243	0.284
s-Biopredsi	0.546	0.296	0.218	0.270
Thermocomposition21	0.577	0.200	0.221	0.288
DSIR	0.555	0.158	0.222	0.295
desiRm^21^	0.558	0.164	0.222	0.294

### Increase of siRNA efficacy by base substitution

The siRNA pathway is a multistep procedure and one crucial step is the integration of the guide strand into the RISC complex. The efficiency of integration depends on the sequence of siRNA duplexes, but likely not on the sequence of the target sites itself [20], [30]. Here in this section we propose an ingenious approach to design non-perfect siRNAs, which are more efficient in the earlier steps of the process such as RISC integration resulting more potent siRNAs.

RNAi studies in human cells showed effective siRNAs may have length from 16 to 21 nt [32], siRNA of length of 19 nt have been successfully used to silent mRNAs [23], [24], [27], [33]. Previously, it has been shown that performance of siRNA prediction method developed using 19 nt is very similar to method developed using 21 nt [8]. In order to understand the effect of mismatch between siRNA and target, first time a systematic experimental analysis was conducted by Liang's group [15], [17]. They used 19 nt long siRNA for targeting human CD46 gene (XM_036622) at nucleotides position 604–622. In order to get more insight on single-nucleotide mismatch, same group studied all combinations of base-mismatch across each position on target sites [21]. They employed 20 siRNAs against ∼400 target sites and generate most comprehensive data on efficacy of single mutation on target site. Hence, for implementing the result of these studies we developed a SVM model desiRm^19^. This model uses same nucleotide features as with desiRm^21^ but on 19 nt long sequence, which were made by removing last two bases from 3′-end of each 21 nt long sequence [34]. desiRm^19^ achieved correlation coefficient of 0.646, 0.648, and 0.553 on training dataset, independent datasets of 249- and 419-sequences respectively. The performance of desiRm^19^ is marginally lower than desiRm^21^ because of less information content on 19 nt long sequence.

In past, several investigations reported the importance of target site accessibility in mRNA to design effective siRNA. Hence, we also integrated target site accessibility feature along with nucleotide frequency and binary pattern feature (desiRm^19^) for model development. The best SVM model (desiRm) achieved correlation coefficient of 0.647 and 0.654 on training dataset (2182-sequences) and independent dataset (249-sequences) respectively. The marginal improvement in the performance was observed due to incorporating target site accessibility information. This supports earlier finding about the importance of this feature in designing functional siRNA [6], [35].

In order to get more potent siRNA, we generated mutation on every position of 19 nt antisense with all four nucleotides of an siRNA. Efficacies of these mutated siRNAs were predicted using our SVM model desiRm. However, the mutant siRNA when bound with target sequence caused mismatch and hence affected the silencing efficiency. Therefore, based on the experimental data a scoring method has been developed, which deduced effect of position and/or identity of mismatch from predicted efficacy to find out overall *Mismatch Efficacy* (ME) of mutated siRNA.

ME =  predicted efficacy- Σ reduced efficacy (due to mismatch)

### Mismatch efficacy incorporating both position and identity of nucleotide

Initially we generated the single mutation that makes 57 different permutation of single siRNA. The repression changes affected by position of mismatch and identity of mismatch between siRNA:target is taken from experimental data [21]. We obtained the mismatch tolerance efficacy data by personal communication with author (Figure S4 of [21]). Therefore a mismatch efficacy is calculated by deducing efficacy due to mismatch from predicted efficacy.

**Figure 1:**
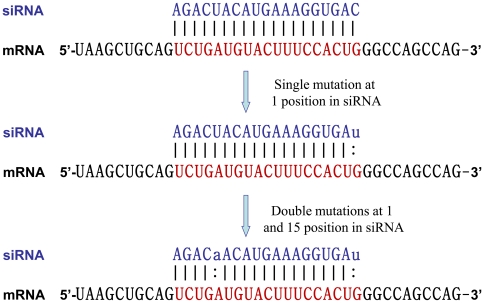
Schematic diagram of efficacy of complementary and mismatch siRNAs against a target site. Fully complementary siRNA has actual and predicted efficacy of 0.479 and 0.588 respectively. Single mutation at 1^st^ position in the siRNA has predicted efficacy of 0.776 but overall efficacy due to single mismatch is 0.710 (0.776-0.066). Further mutation at 15^th^ position in siRNA has predicted mismatch efficacy of 0.929. Base pairing is denoted by “ | ”, mismatch with “ : ”, and mutant base with small case.

Suppose an siRNA (CAGUGGAAAGUACAUCAGA) is made against a target region (UCUGAUGUACUUUCCACUG) in a mRNA NM015213. The siRNA is fully complementary with target having actual and predicted efficacy of 0.479 [4] and 0.588 respectively (Figure 1). If C is replaced by U at 1^th^ position (uAGUGGAAAGUACAUCAGA) its predicted efficacy will be 0.776, when it is fully complementary with target sequence. But as we noticed that first base of siRNA causes mismatch of U:G and causes decrease in efficacy by 0.066 (from [21]). Thus mismatch efficacy of uAGUGGAAAGUACAUCAGA is 0.776-0.066  =  0.710. To find out efficacy of siRNA with two mismatches experimental data were taken from Dahlgren *et al.,* [17]. Thus, subsequent mutations resulting siRNA of uAGUGGAAAGUACAaCAGA with mismatch efficacy of 0.929.

### Mismatch efficacy incorporating only position effect

The experimental studies carried out by Dahlgren *et al.* only used single siRNA against mutated targets [17]. Thus, the effects of all possible types of siRNAs and double nucleotides mismatches were not studied. The experimental data has 709 different combinations for double nucleotide mismatch out of 1539 possible. Therefore, in case of more than two mismatches or lack of similarity with experimental data we only incorporated average position specific effect from single-nucleotide mismatch [21]. For position specific mismatch, average effect of that position was considered (Figure S1).

### Description of web server

A user-friendly webserver has been developed on SUN server under Solaris environment using HTML, PERL, and CGI-PERL. There are two input fields; **(1) submit mRNA:** effective siRNA can be detected against the mRNA (Figure S2). The output result is in descending order of efficacy that contains sequences of antisense with fully complementary target sequence, position in mRNA, target site accessibility and its efficacy (Figure S3). If one wants to further increase the potency of siRNA then more efficacious antisense sequence can be clicked which is submitted automatically to generate single mutant siRNAs and rank them according to ME efficacy (Figure S4). The output result shows the position of mismatch, mutated nucleotide, target site accessibility as well as targets sequence. The increase in efficacy using mutation can also be obtained directly by using second input field, **(2) submit siRNA**, where user can put its 19 nt long antisense siRNA generated from other software and its target sequence. However, this field did not consider the target site accessibility feature during efficacy calculation. Further mutations in siRNA can be generated by clicking the antisense sequence. This strategy can also be used to generate siRNA with very low efficacy against an off-target.

### Comparison of efficacy due to mismatch

An analysis was carried out to assess the effect on efficacy due to mismatch between siRNA and target sequence. We considered mutant siRNAs sequence against a particular target. By using our server it was found that 1–2 mutation can be use to reverse the efficacy of a siRNA from ineffective to effective and vice-versa but need experimental verification (Table 4). Therefore, in order to evaluate real performance of desiRm, we evaluated its performance on experimentally verified 78 mutated siRNA, taken from Ohnishi *et al.* [36]. In this study, they design allele specific siRNA to degrade mutant mRNA of human Prion Protein (PRNP) gene without depleting wild type transcript (Figure S5). They utilized same strategy which we are proposing, *i.e.* targeting same site with different siRNAs (each siRNA having one-base substitution at different position) to manipulate the efficacy of siRNA and to get those siRNAs which can better discriminate between mutant and wild type target. Thus siRNA give rise to single-nucleotide mismatch with mutant-target while two-nucleotide mismatch with wild-type. They reported that introducing base-substitution at specific position in siRNA depleted the mutant transcript while least affected on wild-type. When we predict the efficacy of siPrnp 102 (T9) by desiRm a correlation coefficient of 0.725 was achieved between actual and predicted efficacy (Table 5). This high correlation supports the applicability of our tool in real life. Furthermore, we also used desiRm on another set of siRNA data and achieved correlation coefficient of 0.586, 0.607 and 0.666 between actual and predicted efficacy for siPrnp105(T10), siPrnp102(T10) and siPrnp178(A9) respectively (See Table S2, S3, S4).

**Table 4:** Comparative study of increase/decrease efficacy of siRNAs by using our method, desiRm.

siRNA antisense	Target access	Actual Efficacy	desiRm Efficacy	Mutated siRNA antisense	desiRm Efficacy	Position of Mutation
UCCUCACCAUCCGUCCAGU	0.003895	0.465	0.577	UCCUCACCcUCCGUCCAGg	0.771	9, 19
CUAAUAUGUUAAUUGAUUU	0.054683	0.462	0.647	CUAAUAUGUUAAUUGAUUg	0.813	19
				CUAAUAUcUUAAUUGAUUg	0.855	8,19
				uUAAUAUGUUAAUUGAUUg	0.909	1,19
CAGAUUCCACACCAUGUGG	0.000327	0.402	0.732	uAGAUUCCACACCAUGUGG	0.864	1
				aAGAUUCCACACCAUGUGG	0.923	1
				uAGAUUCCACACCAaGUGG	1.033	1,15
				uAGAUUCCACACCAcGUGG	0.148	1, 15
				uAGAUcCCACACCAUGUGG	0.061	1,6
GGUCCACAUUCUAUUUUAA	0.007570	0.388	0.397	aGUCCACAUUCUAUUUUAA	0.628	1
				uGUCCACAUUCUAUUUUAg	0.798	1, 19
				uGUCCACAUUCUAUUUUcg	0.757	1, 18, 19
CCUCACCAUCCGUCCAGUA	0.002853	0.326	0.473	aCUCACCAUCCGUCCAGUA	0.653	1
				uCUCACCAUCCGUCCAGUg	0.760	1, 19
UGUCUACAAUCCACUGUGU	0.008437	0.993	0.878	UGUCUACAAaCCACUGUGU	0.188	10
				UGUCUACAuUCCACUGUGU	0.038	9
AACUUCUUGGCUUUGUACU	0.023926	0.995	0.895	AACUUCUUGuCUUUGUACU	0.228	10
AACAGCUCCGGAUUCUGUG	0.000321	0.978	0.926	AACAGCUCCGGAUaCUGUG	0.273	14
				AACAGCUCCcGAUUCUGUG	0.260	10
				AACAGCUCCGGAUUaUGUG	0.189	15
UAGAAAUGCACACAUCACC	0.001601	0.947	1.019	UAGAAAUGCACAaAUCACC	0.343	13
AAAACUUCACUACAAAUUC	0.008497	0.967	0.914	AAAACUUCuCUACAAAUUC	0.083	9
				AAAACUUCAaUACAAAUUC	0.027	10

Sequence taken from Huesken data, mutated nucleotide is denotes in lower case. Target access: probability of being unpaired at target site calculated by RNAplfold.

**Table 5:** Assessment of desiRm on experimentally verified mismatched siRNAs of siPrnp102(T9).

Name of siRNA	siRNA sequence (antisense) Mutated sequence	# Mismatch (mRNA)	Target sequence	siRNA:Target (base mismatch position on siRNA)	Actual Efficacy	Predicted efficacy
siPrnp102(T9)	UGGCUUACUCAGCUUGUUC	0 (mutant)	GAACAAGCUGAGUAAGCCA	0	0.972	0.942
siPrnp102(T9)-5U	UGGCUUACUCAGCU**a**GUUC	1(mutant)	GAAC**A**AGCUGAGUAAGCCA	A:A(15)	0.953	0.199
siPrnp102(T9)-6U	UGGCUUACUCAGC**a**UGUUC	1(mutant)	GAACA**A**GCUGAGUAAGCCA	A:A(14)	0.864	0.267
siPrnp102(T9)-7C	UGGCUUACUCAG**g**UUGUUC	1(mutant)	GAACAA**G**CUGAGUAAGCCA	G:G(13)	0.867	0.531
siPrnp102(T9)-12C	UGGCUUA**g**UCAGCUUGUUC	1(mutant)	GAACAAGCUGA**G**UAAGCCA	G:G(8)	0.931	0.645
siPrnp102(T9)-13A	UGGCUU**u**CUCAGCUUGUUC	1(mutant)	GAACAAGCUGAG**U**AAGCCA	U:U(7)	0.951	0.821
siPrnp102(T9)-14U	UGGCU**a**ACUCAGCUUGUUC	1(mutant)	GAACAAGCUGAGU**A**AGCCA	A:A(6)	0.949	0.571
siPrnp102(T9)-15U	UGGC**a**UACUCAGCUUGUUC	1(mutant)	GAACAAGCUGAGUA**A**GCCA	A:A(5)	0.964	0.720
siPrnp102(T9)-16C	UGG**g**UUACUCAGCUUGUUC	1(mutant)	GAACAAGCUGAGUAA**G**CCA	G:G(4)	0.850	0.664
siPrnp102(T9)-17G	UG**c**CUUACUCAGCUUGUUC	1(mutant)	GAACAAGCUGAGUAAG**C**CA	C:C(3)	0.941	0.782
siPrnp102(T9)	UGGCUUACUC**a**GCUUGUUC	1 (wt)	GAACAAGC**C**GAGUAAGCCA	A:G (11)	0.763	0.450
siPrnp102(T9)-5U	UGGCUUACUC**a**GCU**a**GUUC	2 (wt)	GAAC**A**AGC**C**GAGUAAGCCA	A:A(15)/A:G (11)	0.513	0.150
siPrnp102(T9)-6U	UGGCUUACUC**a**GC**a**UGUUC	2 (wt)	GAACA**A**GC**C**GAGUAAGCCA	A:A(14)/A:G (11)	0.403	0.134
siPrnp102(T9)-7C	UGGCUUACUC**a**G**g**UUGUUC	2 (wt)	GAACAA**G**C**C**GAGUAAGCCA	G:G(13)/A:G (11)	0.400	0.033
siPrnp102(T9)-12C	UGGCUUA**g**UC**a**GCUUGUUC	2 (wt)	GAACAAGC**C**GA**G**UAAGCCA	G:G(8)/A:G (11)	-0.041	0.143
siPrnp102(T9)-13A	UGGCUU**u**CUC**a**GCUUGUUC	2 (wt)	GAACAAGC**C**GAG**U**AAGCCA	U:U(7)/A:G (11)	0.183	0.286
siPrnp102(T9)-14U	UGGCU**a**ACUC**a**GCUUGUUC	2 (wt)	GAACAAGC**C**GAGU**A**AGCCA	A:A(6)/A:G (11)	-0.135	0.176
siPrnp102(T9)-15U	UGGC**a**UACUC**a**GCUUGUUC	2 (wt)	GAACAAGC**C**GAGUA**A**GCCA	A:A(5)/A:G (11)	0.388	0.217
siPrnp102(T9)-16C	UGG**g**UUACUC**a**GCUUGUUC	2 (wt)	GAACAAGC**C**GAGUAA**G**CCA	G:G(4)/A:G (11)	0.126	0.265
siPrnp102(T9)-17G	UG**c**CUUACUC**a**GCUUGUUC	2 (wt)	GAACAAGC**C**GAGUAAG**C**CA	C:C(3)/A:G (11)	-0.063	0.178

siPrnp102(T9) and its various mutant siRNAs were targeted against prion protein genes (PRNP) and its mutant allele (PRNP-P102L). Mutated base in siRNA is denoted by small letter while mismatch base between siRNA and target are denoted by bold letter. Data of actual efficacy of siRNAs were taken from experimental work reported by Ohnishi *et al* [36]. Predicted efficacy denotes efficacy of desiRm. All sequences are in 5′ to 3′ direction. Correlation coefficient between actual and predicted efficacy is R = **0.725.**

## Discussion

It is well known that final outcome of siRNA efficacy is the contribution of efficacy gain at each step of RNAi pathway from loading of guide strand into RISC, target accessibility, and cleavage efficiency [23], [30], [37], [38]. However, their degree of contribution is not fully known. Taken together these studied indicate that there are rooms to make mutations in siRNA which become more accessible to different proteins involved in RNAi pathway to enhance the silencing effect. In past, various regression methods were developed to predict the efficacy of siRNA using large experimental data. But there is lack of method that can design the highly effective siRNA by generating mismatch between siRNA and target sequence. The principle of our method is to design siRNAs, which gain efficacy at various steps of RNAi pathways and at last step, silencing, incorporate the mismatch effect with target site.

Here first we have developed robust SVM model for efficacy prediction of siRNA using nucleotide features. Although we got similar performance of our method, desiRm, as other methods but extensive improvement of performance was not possible even using other various nucleotide features. Several studies indicated that target site accessibility can improve the siRNA efficacy [6], [35], [39]. Thus we integrated the target site accessibility feature along with nucleotide features and achieved marginally better performance of model. This final model was implemented with mismatched-tolerance data. In the mismatch efficacy prediction we have incorporated both position as well as identity of nucleotide for single, double-nucleotide mismatch taken from experimental data [17], [21]. Dahlgren *et al.* only used single siRNA in their study, thus all possible combination of siRNA and double-nucleotide mismatch was not covered. Therefore, in case of more than two mismatches or lack of similarity with experimental data we only incorporate average position specific effect from single-nucleotide mismatch [21]. A previous method developed specificity score to find out off-target genes but only considered position specific effect from single-nucleotide mismatch data from Du *el al.* [15], [22]. However, Du *et al.* studied the effect of 57 combinations of mismatch while 219 combinations of mismatched out of 228 was covered by Huang *et al.* across all target position [15], [21]. Thus we implemented most comprehensive data of Huang *et al.* in desiRm. Several studies showed the importance of mismatch siRNA for targeting disease associated SNP genes without effecting the normal gene [20], [21], [36], [40], [41]. Performance of our method on experimental data showed better correlation coefficient on mismatch efficacy (R = 0.725) than that of SVM model (R = 0.647) indicating usefulness of desiRm for predicting mutant siRNA.

### Conclusions

In this study we have developed a method to design siRNA against fully complementary as well as partial complementary region. This novel method helps to make siRNA of desired efficacy without changing the target site. This is very important because some region in mRNA can be best candidate because of having least similarity with non-intended mRNA but at same time having lowest efficacy. Furthermore, our method helpful to design siRNA against SNP associated disease causing gene and mutation prone virus like HIV.

## Supporting Information

Figure S1 **Position specific effect on efficacy due to single-nucleotide mismatch.** Position 1,2,3, 18 and 19 were highly tolerable *i.e*. efficacy is least affected.
(PDF)

Figure S2 **Snapshot of desiRm input field where mRNA can be submitted to get siRNAs.**
(JPG)

Figure S3 **Snapshot of desiRm output result with fully complementary siRNAs.** Each row contains sequence of siRNA, target position, target sequence and accessibility with predicted efficacy. To improve the efficacy of 197^th^ siRNA targeting on 164^th^ position (highlighted), click this sequence.
(JPG)

Figure S4 **Snapshot of desiRm output result with single-mutated siRNAs.** Each row contains mutated siRNA, position of mutation, type of mutation, target sequence and accessibility, with predicted efficacy. First sequence (WT) is original, mutation at 1^st^ position in siRNA increase their efficacy to 0.710. Further improvement could be achieved by click on siRNA.
(JPG)

Figure S5 **Complete CDS of** ***Homo sapiens*** **prion protein (PRNP) gene (wild type).** The nucleotides in bold and red color indicate the position of nucleotide variation in mutant genes reported. Mutant PRNP-P102L has mutation at position 377(C→U); mutant PRNP-P105L has mutation at position 386(C→U); mutant PRNP-D178N has mutation at position 564(G→A). Highlighted regions are targeted by siRNAs in both wild type and mutants by Ohnishi et al.
(PDF)

Table S1 **Performance of SVM-based model for siRNA efficacy prediction developed using hybrid of features.**
(DOCX)

Table S2 **Assessment of desiRm on experimentally verified mismatched siRNAs of siPrnp105(T10).** siPrnp105(T10) and its various mutant siRNAs were targeted against prion protein genes (PRNP) and its mutant allele (PRNP-P105L). Mutated base in siRNA is denoted by small letter while mismatch base between siRNA and target are denoted by bold letter. Data of actual efficacy of siRNAs were taken from experimental work reported by Ohnishi *et al*. Predicted efficacy denotes efficacy of desiRm. All sequences are in 5′ to 3′ direction. Correlation coefficient between actual and predicted efficacy is R = 0.586.
(DOCX)

Table S3 **Assessment of desiRm on experimentally verified mismatched siRNAs of siPrnp102(T10).** siPrnp102(T10) and its various mutant siRNAs were targeted against prion protein genes (PRNP) and its mutant allele (PRNP-P102L). Mutated base in siRNA is denoted by small letter while mismatch base between siRNA and target are denoted by bold letter. Data of actual efficacy of siRNAs were taken from experimental work reported by Ohnishi *et al*. Predicted efficacy denotes efficacy of desiRm. All sequences are in 5′ to 3′ direction. Correlation coefficient between actual and predicted efficacy is R = 0.607.
(DOCX)

Table S4 **Assessment of desiRm on experimentally verified mismatched siRNAs of siPrnp178(A9).** siPrnp178(A9) and its various mutant siRNAs were targeted against prion protein genes (PRNP) and its mutant allele (PRNP-D178N). Mutated base in siRNA is denoted by small letter while mismatch base between siRNA and target are denoted by bold letter. Data of actual efficacy of siRNAs were taken from experimental work reported by Ohnishi *et al*. Predicted efficacy denotes efficacy of desiRm. All sequences are in 5′ to 3′ direction. Correlation coefficient between actual and predicted efficacy is R = 0.666.
(DOCX)
