# Supplementary figures and images for: Designing of Highly Effective Complementary and Mismatch siRNAs for Silencing a Gene
Source: PLoS One. 2011 Aug 10;6(8):e23443. doi: 10.1371/journal.pone.0023443 (PMC3154470; doi:10.1371/journal.pone.0023443)

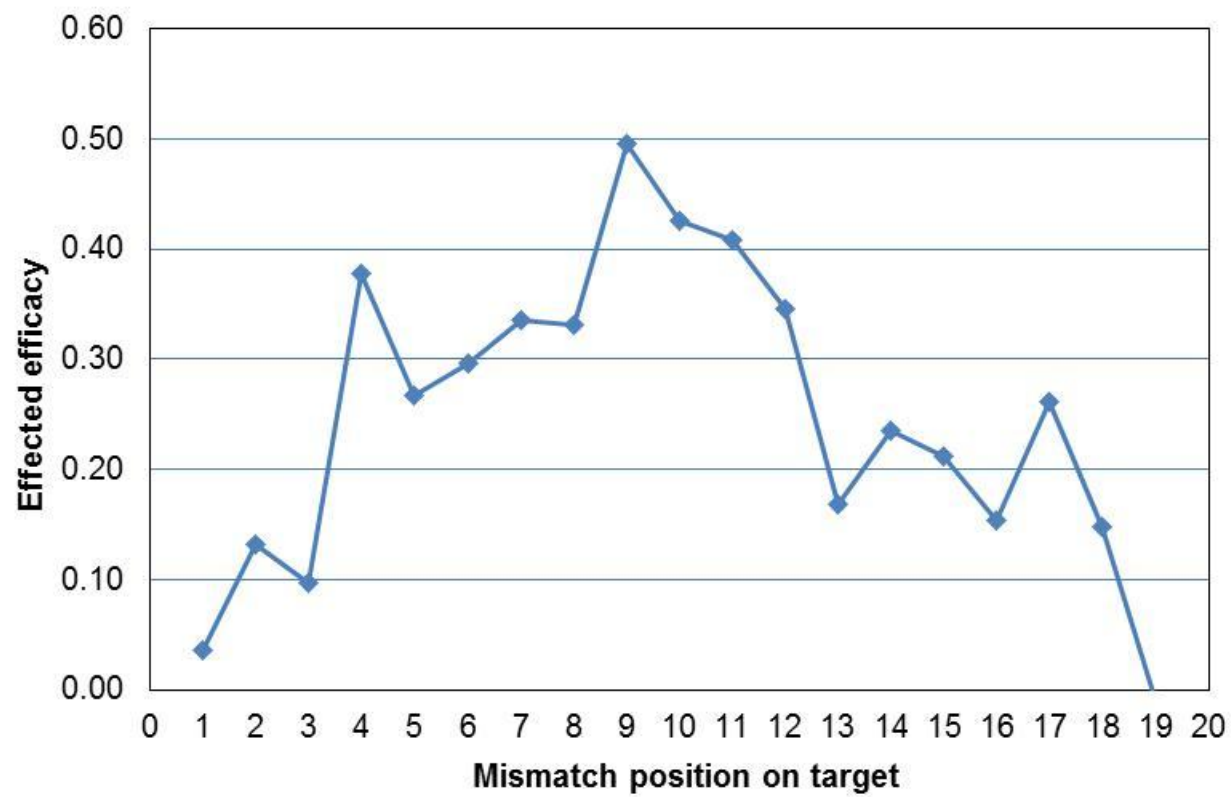

Supplement: Figure S1 — Position specific effect on efficacy due to single-nucleotide mismatch. Position 1,2,3, 18 and 19 were highly tolerable i.e. efficacy is least affected. (PDF) [file pone.0023443.s001.pdf]

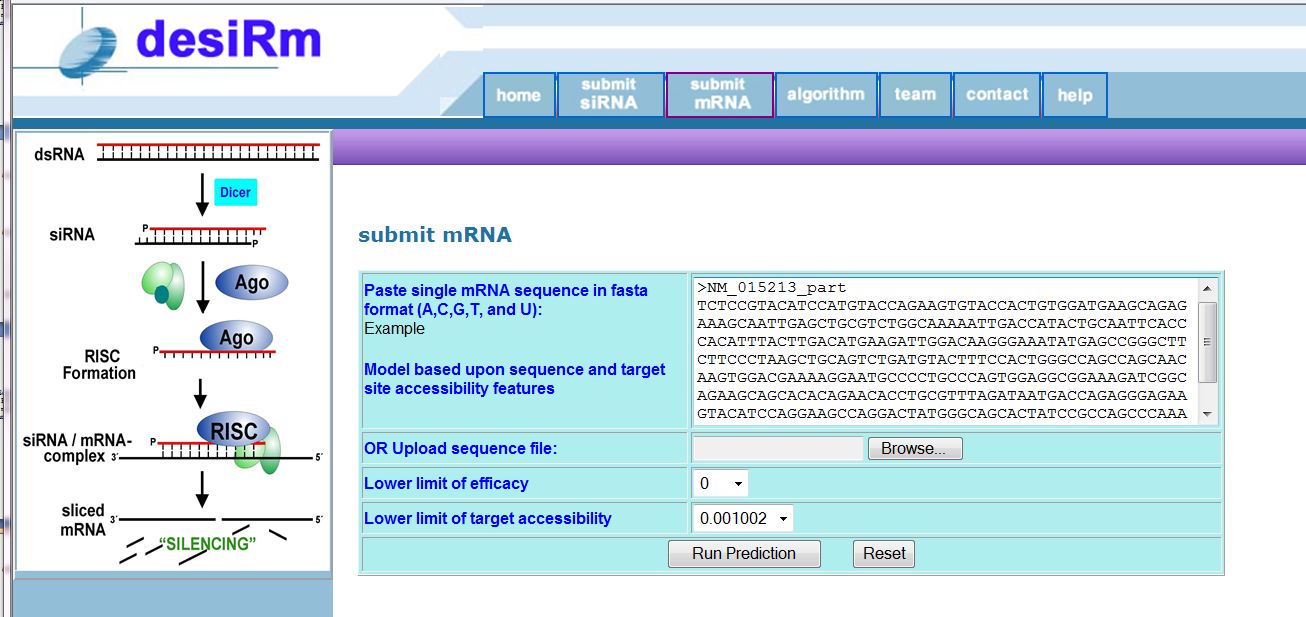

Supplement: Figure S2 — Snapshot of desiRm input field where mRNA can be submitted to get siRNAs. (JPG) [file pone.0023443.s002.jpg]

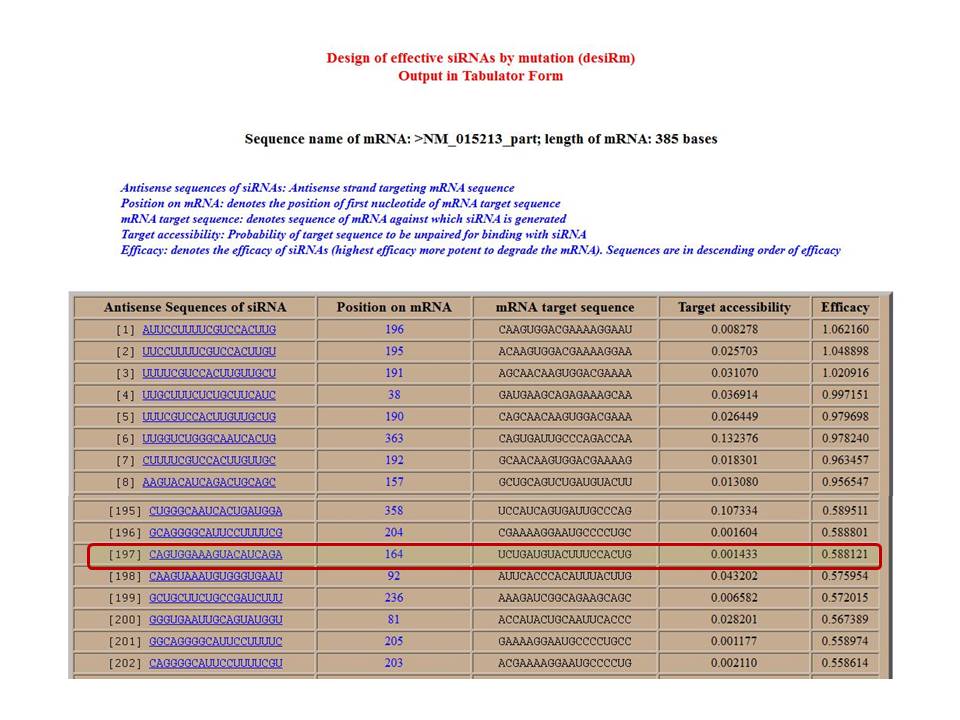

Supplement: Figure S3 — Snapshot of desiRm output result with fully complementary siRNAs. Each row contains sequence of siRNA, target position, target sequence and accessibility with predicted efficacy. To improve the efficacy of 197th siRNA targeting on 164th position (highlighted), click this sequence. (JPG) [file pone.0023443.s003.jpg]

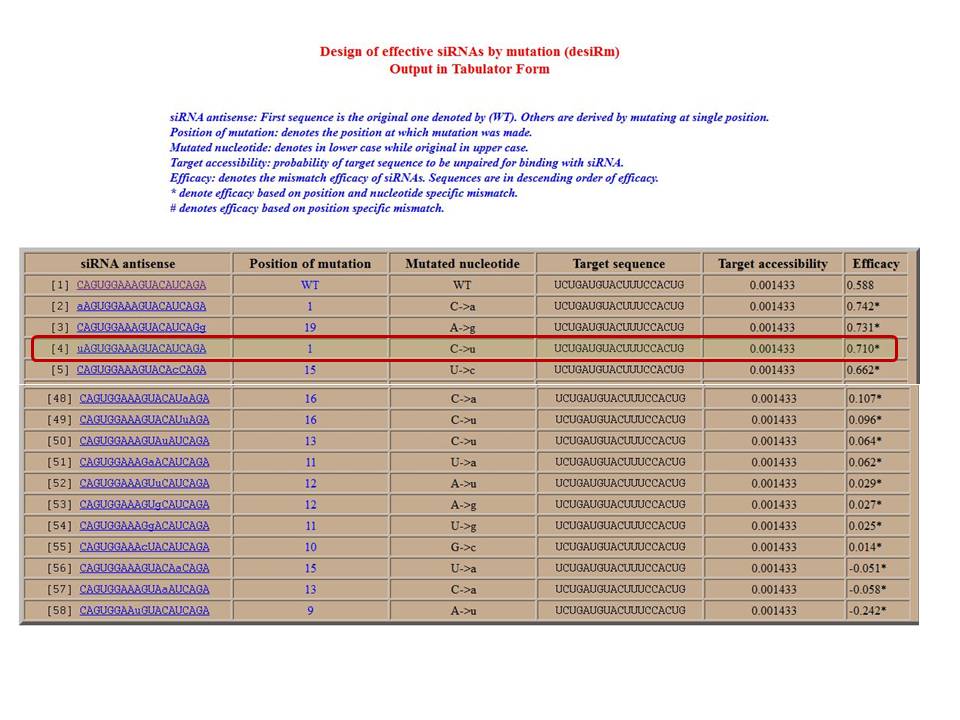

Supplement: Figure S4 — Snapshot of desiRm output result with single-mutated siRNAs. Each row contains mutated siRNA, position of mutation, type of mutation, target sequence and accessibility, with predicted efficacy. First sequence (WT) is original, mutation at 1st position in siRNA increase their efficacy to 0.710. Further improvement could be achieved by click on siRNA. (JPG) [file pone.0023443.s004.jpg]
