# Supplementary material for: Designing of Highly Effective Complementary and Mismatch siRNAs for Silencing a Gene
Source: PLoS One. 2011 Aug 10;6(8):e23443. doi: 10.1371/journal.pone.0023443 (PMC3154470; doi:10.1371/journal.pone.0023443)
Supplement: Figure S5 — Complete CDS of Homo sapiens prion protein (PRNP) gene (wild type). The nucleotides in bold and red color indicate the position of nucleotide variation in mutant genes reported. Mutant PRNP-P102L has mutation at position 377(C→U); mutant PRNP-P105L has mutation at position 386(C→U); mutant PRNP-D178N has mutation at position 564(G→A). Highlighted regions are targeted by siRNAs in both wild type and mutants by Ohnishi et al. (PDF) [file pone.0023443.s005.pdf]

>gi|38490001|gb|AY458651.1| Homo sapiens prion protein (PRNP) gene, complete cds

ATGGCGAACCTTGGCTGCTGGATGCTGGTTCTCTTTGTGGCCACATGGAGTGACCTGGGCCTCTGCAAGA
AGCGCCCGAAGCCTGGAGGATGGAACACTGGGGGCAGCCGATACCCGGGGCAGGGCAGCCCTGGAGGCAA
CCGCTACCCACCTCAGGGCGGTGGTGGCTGGGGGCAGCCTCATGGTGGTGGCTGGGGGCAGCCTCATGGT
GGTGGCTGGGGGCAGCCTCATGGTGGTGGCTGGGGACAGCCTCATGGTGGTGGCTGGGGGCAGCCTCATG
GTGGTGGCTGGGGGCAGCCCCATGGTGGTGGCTGGGGACAGCCTCATGGTGGTGGCTGGGGTCAAGGAGG
TGGCACCCACAGTCAGTGGAACAAGC**C**GAGTAAGC**C**AAAAACCAACATGAAGCACATGGCTGGTGCTGCA
GCAGCTGGGGCAGTGGTGGGGGGCCTTGGCGGCTACATGCTGGGAAGTGCCATGAGCAGGCCCATCATAC
ATTTCGGCAGTGACTATGAGGACCGTTACTATCGTGAAAACATGCACCGTTACCCCAACCAAGTGTACTA
CAGGCCCATGGATGAGTACAGCAACCAGAACAACTTTGTGCAC**G**ACTGCGTCAATATCACAATCAAGCAG
CACACGGTCACCACAACCACCAAGGGGGAGAACTTCACCGAGACCGACGTTAAGATGATGGAGCGCGTGG
TTGAGCAGATGTGTATCACCCAGTACGAGAGGGAATCTCAGGCCTATTACCAGAGAGGATCGAGCATGGT
CCTCTTCTCCTCTCCACCTGTGATCCTCCTGATCTCTTTCCTCATCTTCCTGATAGTGGGATGA
