# Supplementary material for: Designing of Highly Effective Complementary and Mismatch siRNAs for Silencing a Gene
Source: PLoS One. 2011 Aug 10;6(8):e23443. doi: 10.1371/journal.pone.0023443 (PMC3154470; doi:10.1371/journal.pone.0023443)
Supplement: Table S1 — Performance of SVM-based model for siRNA efficacy prediction developed using hybrid of features. (DOCX) [file pone.0023443.s006.docx]

**Table S1 Performance of SVM-based model for siRNA efficacy prediction developed using hybrid of features.**

| **Features** | **Vector** | **R** | **R2** | **MAE** | **RMSE** | **g** | **c** | **j** |
| --- | --- | --- | --- | --- | --- | --- | --- | --- |
| Mono+Dinuc | 20 | 0.520 | 0.230 | 0.137 | 0.175 | 0.001 | 2 | 2 |
| Mono+Trinuc | 68 | 0.568 | 0.317 | 0.131 | 0.165 | 0.001 | 1 | 2 |
| Dinuc+Trinuc | 80 | 0.542 | 0.286 | 0.134 | 0.169 | 0.001 | 2 | 2 |
| Mono+Dinuc+Trinuc | 84 | 0.559 | 0.310 | 0.131 | 0.166 | 0.001 | 2 | 1 |
| Dinuc + 2^nd^ Dinuc + 3^rd^ Dinuc | 48 | 0.524 | 0.270 | 0.136 | 0.171 | 0.001 | 1 | 1 |
| Dinuc + 3^rd^ Dinuc | 32 | 0.504 | 0.235 | 0.139 | 0.175 | 0.001 | 1 | 1 |
| 3^rd^ Dinuc + 2^nd^ Trinuc | 80 | 0.567 | 0.320 | 0.131 | 0.165 | 0.001 | 1 | 1 |
| 3^rd^ Dinuc + 2^nd^ Trinuc [1,10] | 80 | 0.629 | 0.395 | 0.123 | 0.155 | 0.001 | 2 | 1 |
| Binary pattern + Binary of dinuc | 420 | 0.634 | 0.361 | 0.126 | 0.160 | 0.001 | 2 | 2 |
| Binary pattern + Binary of dinuc + Binary of Condense | 460 | 0.628 | 0.393 | 0.123 | 0.156 | 0.001 | 1 | 1 |
| Hydrogen bond + Binary pattern | 105 | 0.640 | 0.393 | 0.124 | 0.156 | 0.01 | 1 | 2 |
| Binary pattern + Thermodynamics | 103 | 0.649 | 0.420 | 0.120 | 0.152 | 0.001 | 7 | 1 |
