# Supplementary material for: Designing of Highly Effective Complementary and Mismatch siRNAs for Silencing a Gene
Source: PLoS One. 2011 Aug 10;6(8):e23443. doi: 10.1371/journal.pone.0023443 (PMC3154470; doi:10.1371/journal.pone.0023443)
Supplement: Table S2 — Assessment of desiRm on experimentally verified mismatched siRNAs of siPrnp105(T10). siPrnp105(T10) and its various mutant siRNAs were targeted against prion protein genes (PRNP) and its mutant allele (PRNP-P105L). Mutated base in siRNA is denoted by small letter while mismatch base between siRNA and target are denoted by bold letter. Data of actual efficacy of siRNAs were taken from experimental work reported by Ohnishi et al. Predicted efficacy denotes efficacy of desiRm. All sequences are in 5′ to 3′ direction. Correlation coefficient between actual and predicted efficacy is R = 0.586. (DOCX) [file pone.0023443.s007.docx]

**Table S2 Assessment of desiRm on experimentally verified mismatched siRNAs of siPrnp105(T10).** siPrnp105(T10) and its various mutant siRNAs were targeted against prion protein genes (PRNP) and its mutant allele (PRNP-P105L). Mutated base in siRNA is denoted by small letter while mismatch base between siRNA and target are denoted by bold letter. Data of actual efficacy of siRNAs were taken from experimental work reported by Ohnishi *et al*. Predicted efficacy denotes efficacy of desiRm. All sequences are in 5’ to 3’ direction. Correlation coefficient between actual and predicted efficacy is R=0.586.

| **Name of siRNA** | **siRNA sequence (antisense) Mutated sequence** | **# Mismatch**  **(mRNA)** | **Target sequence** | **siRNA:Target**  **(base mismatch position on siRNA)** | **Actual Efficacy** | **Predicted efficacy** |
| --- | --- | --- | --- | --- | --- | --- |
| siPrnp105(T10) WT | UUGGUUUUUAGCUUACUCG | 0 (mutant) | CGAGUAAGCUAAAAACCAA | 0 | 0.968 | 1.087 |
| siPrnp105(T10)-5A | UUGGUUUUUAGCUU**u**CUCG | 1(mutant) | CGAG**U**AAGCUAAAAACCAA | U:U(15) | 0.566 | 0.701 |
| siPrnp105(T10)-6U | UUGGUUUUUAGCU**a**ACUCG | 1(mutant) | CGAGU**A**AGCUAAAAACCAA | A:A(14) | 0.500 | 0.444 |
| siPrnp105(T10)-8C | UUGGUUUUUAG**g**UUACUCG | 1(mutant) | CGAGUAA**G**CUAAAAACCAA | G:G(13) | 0.732 | 0.971 |
| siPrnp105(T10)-12U | UUGGUUU**a**UAGCUUACUCG | 1(mutant) | CGAGUAAGCUA**A**AAACCAA | A:A(8) | 0.620 | 0.651 |
| siPrnp105(T10)-13U | UUGGUU**a**UUAGCUUACUCG | 1(mutant) | CGAGUAAGCUAA**A**AACCAA | A:A(7) | 0.442 | 0.860 |
| siPrnp105(T10)-14U | UUGGU**a**UUUAGCUUACUCG | 1(mutant) | CGAGUAAGCUAAA**A**ACCAA | A:A(6) | 0.637 | 0.719 |
| siPrnp105(T10)-15U | UUGG**a**UUUUAGCUUACUCG | 1(mutant) | CGAGUAAGCUAAAA**A**CCAA | A:A(5) | 0.832 | 0.898 |
| siPrnp105(T10)-16U | UUG**a**UUUUUAGCUUACUCG | 1(mutant) | CGAGUAAGCUAAAAA**C**CAA | A:C(4) | 0.665 | 1.133 |
| siPrnp105(T10)-17U | UU**a**GUUUUUAGCUUACUCG | 1(mutant) | CGAGUAAGCUAAAAAC**C**AA | A:C(3) | 0.591 | 0.860 |
| siPrnp105(T10) WT | UUGGUUUUU**u**GCUUACUCG | 1 (wt) | CGAGUAAGC**C**AAAAACCAA | U:U(10) | 0.814 | 0.861 |
| siPrnp105(T10)-5A | UUGGUUUUU**u**GCUU**u**CUCG | 2 (wt) | CGAG**U**AAGC**C**AAAAACCAA | U:U(15)/U:U(10) | 0.197 | 0.332 |
| siPrnp105(T10)-6U | UUGGUUUUU**u**GCU**a**ACUCG | 2 (wt) | CGAGU**A**AGC**C**AAAAACCAA | A:A(14)/U:U(10) | 0.116 | 1.114 |
| siPrnp105(T10)-8C | UUGGUUUUU**u**G**g**UUACUCG | 2 (wt) | CGAGUAA**G**C**C**AAAAACCAA | G:G(13)/U:U(10) | 0.289 | 0.225 |
| siPrnp105(T10)-12U | UUGGUUU**a**U**u**GCUUACUCG | 2 (wt) | CGAGUAAGC**C**A**A**AAACCAA | A:A(8)/U:U(10) | 0.434 | 0.262 |
| siPrnp105(T10)-13U | UUGGUU**a**UU**u**GCUUACUCG | 2 (wt) | CGAGUAAGC**C**AA**A**AACCAA | A:A(7)/U:U(10) | -0.165 | 0.426 |
| siPrnp105(T10)-14U | UUGGU**a**UUU**u**GCUUACUCG | 2 (wt) | CGAGUAAGC**C**AAA**A**ACCAA | A:A(6)/U:U(10) | -0.847 | 0.316 |
| siPrnp105(T10)-15U | UUGG**a**UUUU**u**GCUUACUCG | 2 (wt) | CGAGUAAGC**C**AAAA**A**CCAA | A:A(5)/U:U(10) | -0.073 | 0.393 |
| siPrnp105(T10)-16U | UUG**a**UUUUU**u**GCUUACUCG | 2 (wt) | CGAGUAAGC**C**AAAAA**C**CAA | A:C(4)/U:U(10) | 0.415 | 0.496 |
| siPrnp105(T10)-17U | UU**a**GUUUUU**u**GCUUACUCG | 2 (wt) | CGAGUAAGC**C**AAAAAC**C**AA | A:C(3)/U:U(10) | 0.247 | 0.436 |
